# Opportunities and challenges for scientific exchange in the Baltic Sea region: Lessons from the Baltic conferences on obstetrics and gynecology (1987–2001)

**DOI:** 10.1111/aogs.14383

**Published:** 2022-05-23

**Authors:** Nils Hansson

**Affiliations:** ^1^ Department for the History, Philosophy, and Ethics of Medicine, Centre for Health and Society, Medical Faculty Heinrich‐Heine‐University Duesseldorf Duesseldorf Germany

Obstetricians and gynecologists in the Baltic Sea region collaborate with colleagues in neighboring countries in many ways, not least through participation at the congresses of the Nordic Federation of Societies of Obstetrics and Gynecology (NFOG).^1^ What did Baltic networks in obstetrics and gynecology look like during the Cold War and its aftermath?^2^ Drawing on oral history with participants of “The Baltic Conferences on Obstetrics and Gynecology”, which took place between 1987 and 2001, and on conference proceedings, I will try to reconstruct challenges colleagues faced when they collaborated across the iron curtain and during the first decade after the Cold War. Using these conferences as a lens, I wish to add a perspective to the current debate about cooperation between physicians in northern Europe during turbulent political times.

The Baltic Conferences on Obstetrics and Gynecology were launched by, among others, Stig Kullander (Malmö, Sweden) and Kurt Semm (Kiel, Germany) in January 1986, but the original idea was proposed by the Southern Swedish Gynecological Society already in the early 1980s. The first conference took place at the Wilhelm Pieck University in Rostock, German Democratic Republic (GDR), from May 22 to 24, 1987. Hans Wilken, the president of the conference, emphasized in his greeting that the meetings should promote the exchange of knowledge between gynecologists in the Baltic Sea area: “The idea of a Baltic conference is to bring together obstetricians and gynecologists […] living in the area of the Baltic Sea.”^3^ While there were only 16 scientific presentations at the first conference in 1987, the network had already expanded considerably in 1989, when the abstract book included more than 60 presentations and 229 senior and junior participants from all countries around the Baltic Sea.

Between 1987 and 2001, a series of eight conferences was held in eight countries, all sponsored by pharmaceutical companies, with scientific sessions and social program (1987 Rostock‐Warnemünde, 1989 Gdańsk, 1991 Tallinn‐Tartu, 1993 Turku, 1995 Malmö, 1997 Kiel, 1999 Sankt Petersburg, 2001 Riga). A wide spectrum of women's health issues were covered as demonstrated by the key topics presented at two conferences: In 1987 focus was on in vitro fertilization, Gonadotropin releasing hormones, preeclampsia, cesarean section, and future possibilities in pelviscopy, and in 1993, early detection of endometrial and ovarian cancer, ultrasound and doppler in perinatology, coagulation disorders in obstetrics, antenatal care, modern treatment of breast cancer, surveillance during labor, and long‐acting contraception.

To date, I could interview five organizers, who also participated regularly at the Baltic conferences: Helle Karro, Tartu, Gunta Lazdāne, Riga, Liselotte Mettler, Kiel, Karel Maršál, Malmö, and Nils‐Otto Sjöberg, Malmö (the open access video‐recorded interviews, some held online due to the Covid‐19 pandemic, can be viewed here https://mediathek.hhu.de/playlist/1251). After an introduction about the interviewee (university studies, first research experiences as obstetrician and/or gynecologist, scholarly focus), key questions included what role the scientific exchange from the conferences actually played in research and in everyday clinical practice, how the Baltic collaboration changed after 1990 across the (former) iron curtain, and which factors (barriers and drivers) influenced the transfer of knowledge.^4^ In addition, I gathered conference proceedings and abstract books to get a fuller picture of the scientific activities at the meetings. One valuable “real‐time” account about the collaboration is given in an *Acta Obstetricia et Gynecologica Scandinavica* supplement in 1997 where representatives from Sweden (K. Maršál, N‐O. Sjöberg), Estonia (H. Sinimäe), Latvia (G. Lazdane, E. Melks), Lithuania (A. Venckauskas) and Poland (J. Mielnik) shared their national perspectives on the impact of previous and current scientific exchange via the Baltic conferences and beyond. Prof. Sinimäe of Tartu, for instance, elaborated on the fact that at that time (1997) Estonian researchers and clinicians had stronger ties to Finland, Sweden and Germany than to Latvia and Lithuania. She added that “…contacts with Russian colleagues have become infrequent”.^5^

From the perspective of the interviewees in this oral history project, the participants reflected on the circulation of knowledge on different levels, including joint scientific publications, the exchange of instruments and devices (eg the Swedish delegation gave an ultrasound device to the Polish colleagues at the 1989 Gdansk meeting), textbooks, guest lectures and the arrangement of study trips to clinics around the Baltic Sea. For example, Nils‐Otto Sjöberg mentioned that he – after discussions with Lech Wałęsa – organized study trips for Polish gynecologists to Sweden. Next to such drivers for further collaboration, the interviewees also pointed at barriers, first and foremost limited funding and bureaucratic hurdles regarding travel applications. Not all scholars from Eastern Europe got permission to travel to the West. Another challenge delegates brought up in the interviews was the communication during the conferences, with regards to both linguistics and different scholar traditions. The conference language was English, but according to interviewees not all speakers were skilled enough to present in a clear‐cut way. Some of the delegates also underlined that the Nordic countries were considered as scientifically leading. In addition, eyewitnesses explained that different health systems, academic/structural attitudes, and national scientific styles sometimes made a fruitful exchange difficult: “not all participants had been trained in evidence‐based medicine”, as one participant pointed out.

Having said that, all interviewed delegates agreed that the Baltic conferences all in all contributed to building bridges between nations during difficult political circumstances, and that they expanded the scientific network of the participants. So, why did the meetings then end in 2001? Gunta Lazdāne, president of the 2001 Riga conference, wrote in her welcome greeting: “It is time to decide whether there is a need for so many conferences in the Nordic part of Europe, and we should do it together…”. The interviewees emphasized three reasons why the series of meetings did not continue. First, scholars had by then better possibilities to travel and preferred to visit world conferences instead, predominantly in the United States, to keep track of cutting‐edge research. Second, particularly the Baltic delegations used the *Acta Obstetricia et Gynecologica Scandinavica* to a larger extent as a forum to share research. Third, the chief conference initiators of the meetings did make many new contacts, but for the more junior level scholars the conferences seem not to have created as many new connections as hoped in the beginning. In summary, the eight Baltic Conferences on Obstetrics and Gynecology that took place between 1987 and 2001 raise questions about how we can further strengthen our scientific network around the Baltic Sea. Reconstructing and evaluating the opportunities and challenges from these Baltic Conferences can hopefully give valuable hints on how to deepen the scientific collaboration within the field in northern Europe.

## Funding information

Currently, research about how the circulation of knowledge in medicine played out during the Cold War is conducted by the “Bridging the Baltic network”, which consists of around twenty historians of medicine and Cold War historians in the Baltic Sea region. The network is funded by the German Research Foundation (PI: Nils Hansson).
